# Use of Amperometric and Potentiometric Probes in Scanning Electrochemical Microscopy for the Spatially-Resolved Monitoring of Severe Localized Corrosion Sites on Aluminum Alloy 2098-T351

**DOI:** 10.3390/s21041132

**Published:** 2021-02-06

**Authors:** Rejane M. P. da Silva, Javier Izquierdo, Mariana X. Milagre, Abenchara M. Betancor-Abreu, Isolda Costa, Ricardo M. Souto

**Affiliations:** 1 Instituto de Pesquisas Energéticas e Nucleares—IPEN, CNEN—Av. Prof. Lineu Prestes, 2242 São Paulo, Brazil; mariana.milagre@ipen.br (M.X.M.); icosta@ipen.br (I.C.); 2 Departamento de Química, Universidad de La Laguna, P.O. Box 456, E-38200 La Laguna (Tenerife), Canary Islands, Spain; jizquier@ull.edu.es (J.I.); abetanco@ull.edu.es (A.M.B.-A.); 3 Instituto de Materiales y Nanotecnología, Universidad de La Laguna, P.O. Box 456, E-38200 La Laguna (Tenerife), Canary Islands, Spain

**Keywords:** scanning electrochemical microscopy, amperometric and potentiometric probes, aluminum alloy 2098-T351, severe localized corrosion, Mg-Al galvanic pair

## Abstract

Amperometric and potentiometric probes were employed for the detection and characterization of reactive sites on the 2098-T351 Al-alloy (AA2098-T351) using scanning electrochemical microscopy (SECM). Firstly, the probe of concept was performed on a model Mg-Al galvanic pair system using SECM in the amperometric and potentiometric operation modes, in order to address the responsiveness of the probes for the characterization of this galvanic pair system. Next, these sensing probes were employed to characterize the 2098-T351 alloy surface immersed in a saline aqueous solution at ambient temperature. The distribution of reactive sites and the local pH changes associated with severe localized corrosion (SLC) on the alloy surface were imaged and subsequently studied. Higher hydrogen evolution, lower oxygen depletion and acidification occurred at the SLC sites developed on the 2098-T351 Al-alloy.

## 1. Introduction

The 2098 aluminum alloy belongs to the third generation of Al-Li alloys. These alloys were developed as possible substitutes for traditional 2XXX-series Al alloys for aerospace applications [1], with the aim of reducing aircraft weight and, consequently, fuel consumption. In fact, the addition of Li to these new generation alloys aims to decrease the weight of aircrafts [2,3]. These alloys are reported to have low density, good mechanical strength, and good toughness [4,5,6]. Al-Cu-Li alloys, such as 2098, present a reduction in Li content compared to previous first and second generation alloys [2,7], and this reduction causes greater toughness compared to alloys from previous generations [5,6]. This feature represents a significant advantage, since low toughness characteristics were a disadvantage in previous Al-Li alloys. The 2098 Al-Cu-Li alloy has a chemical composition that contains elements such as Cu, Li, Mg, Mn, Zr, Ag and Ti responsible for its advanced microstructure [7,8]. The main phase responsible for the good mechanical properties of Al-Cu-Li alloys is the T1 phase (Al_2_CuLi) [2,7,9]. However, the corrosion mechanism of Al-Cu-Li alloys is related to the T1 phase and micrometric particles enriched with Cu and Fe [2,3]. Several studies have reported that Al-Cu-Li alloys are susceptible to localized corrosion, with the preferential attack occurring at the T1 phase sites, and this attack is commonly called severe localized corrosion (SLC) [10,11,12,13]. In these studies, the microstructure and corrosion mechanisms related to the SLC have been presented. However, localized electrochemical techniques were not used to monitor the electrochemical activity related to the development of SLC in the Al-Cu-Li alloys investigated in these studies. Therefore, it is expected that scanning probe techniques can provide new insights related to the corrosion activity associated with local reactions that occur on this kind of material.

Chemical imaging of reactive samples with high spatial resolution is available by scanning electrochemical microscopy (SECM) methods. SECM can monitor the reactions that occur at a mobile microelectrode (tip), which scans a nearby surface immersed in an electrolytic solution [14]. SECM has different modes of operation, which are broadly classified as amperometric and potentiometric modes, depending on the type of probe being used, be it an ultramicroelectrode (UME) or an ion selective microelectrode (ISME), respectively [15]. Typically, amperometric microelectrodes are the probes used in corrosion studies with SECM ([16,17,18] and references cited therein), although potentiometric SECM has proven to be a powerful tool in corrosion science, primarily for the study of galvanic corrosion processes [19,20,21]. Furthermore, the combination of amperometric and potentiometric modes using separate individual probes has become an interesting option to obtain new insights into the corrosion mechanisms of different materials [22,23]. Different processes have been studied which involve a pH gradient and the heterogeneous release of ions [24,25,26,27]. For instance, the local variation in pH at the anodic sites due to metal dissolution and hydrolysis, related to the co-dissolution of Zn and Al from the Al-Zn metallic layers, and the cut-edge corrosion on painted metals were monitored using the potentiometric mode [24,25,26]. By combining the amperometric/potentiometric operation modes, the distribution of local anodic and cathodic activities on painted cut edges were observed [28]. It must be noted, that changes in the pH related to the cathodic process and to the hydrolysis of dissolved metals, strongly influence the corrosion rate [29,30]. So far, the amperometric/potentiometric operation has been achieved through the use of separate individual probes that had to be exchanged or attached next to each other, resulting in an imperfect match of the scanned surface. Attempts to overcome this limitation have consisted of using a single probe that could be modified in situ for alternative amperometric and potentiometric operations [23], and more recently, by designing multi-probe assemblies that effectively monitor multiple signals in a single experiment [29,30]. In the first case, a double Pt/Sb probe was employed for quasi-simultaneous amperometric detection and visualization of local changes in pH changes of reactive sites in a corroding system [29]. On the other hand, actual simultaneous operation was achieved using multi-probe assemblies for various potentiometric determinations, such as detection of changes in local pH and Zn ion concentration during the galvanic corrosion of a Zn-Cu system [30].

The objective of this work was to explore the possibilities of SECM to monitor local electrochemical activity and changes in pH associated with the development of reactive sites in the severe localized corrosion (SLC) of aluminum alloy 2098-T351. Although SECM in amperometric operation has been used to study the local corrosion and the corrosion protection of various aluminum alloys in different conditions [31,32,33,34,35,36], the use of SECM for the characterization of reactive sites and to visualize local pH changes in Al–Cu–Li alloys has not been attempted until now. Here, platinum (Pt) and antimony (Sb) microelectrodes were investigated as amperometric and potentiometric probes, respectively, for local electrochemical characterization of reactive sites and detection of pH changes. To address responsiveness, these sensing probes were first used to characterize the galvanic corrosion of a model Mg-Al pair immersed in an aqueous NaCl solution. They were then used for characterization of the 2098-T351 alloy under the same conditions by SECM in amperometric and potentiometric operations.

## 2. Materials and Methods

### 2.1. Samples and Solutions

A magnesium/aluminum galvanic couple was used as the model corroding system. Magnesium ribbon with 200 μm × 800 μm cross section and 99.0% purity was supplied by Panreac AppliChem (ITW Reagents, Glenview, IL, USA), and 99.99% pure aluminum wire 125 μm diameter by Goodfellow (Cambridge, UK). In addition to the nominal composition of magnesium in the ribbon, the substances reported to be insoluble in hydrochloric acid were Cu ≤ 0.005%, Fe ≤ 0.05%, Ni ≤ 0.005%, Pb ≤ 0.002%, and Zn ≤ 0.02%. The two metals were embedded in *Epofix* epoxy resin (Struers, Ballerup, Denmark) with 1 mm distance between them, and cured for 24 h at ambient temperature. Only their cross sections were exposed on the front side of the disk-shaped resin mount (dia. 3 cm), and they extended about 10 mm at the rear for electric connection. The front side of the mount was abraded with a sequence of silicon carbide papers down to 4000 grit, followed by polishing with diamond slurries of 3 and 1 μm particle size. After the surfaces were thoroughly rinsed with Millipore deionized water, dried with ethanol, and finally surrounded laterally by sellotape, creating a container holding approximately 5 mL of the test electrolyte solution. Next, a mount containing a flat sample of the 2098-T351 Al-alloy (wt%: 3.4 Cu, 1.0 Li, 0.3 Mg, 0.3 Ag, 0.4 Zr, 0.04 Fe, 0.05 Si, 0.02 Zn, 0.003 Mn) was produced using the *Epofix* resin, with a working area of approximately 5.0 × 5.0 mm^2^. After curing, the same sequence of surface finishing, cleaning and preparation of a container for the test solution was performed.

Experiments were performed in solutions prepared with analytical grade reagents and ultra-pure deionized water. NaCl (Panreac^®^, Barcelona, Spain) was used to prepare the test aqueous electrolyte solutions employed in this study.

### 2.2. Instrumentation

High-resolution SECM equipment, built by Sensolytics (Bochum, Germany), was employed. The instrument was built around an Autolab (Metrohm, Herisau, Switzerland) electrochemical interface, controlled with a personal computer. Amperometric and potentiometric operations were available in this configuration. Measurements were made at room temperature and the samples were left unpolarized to their spontaneous open circuit or galvanic coupling potentials. Raster scanning was employed to record the consecutive line scans composing the *XY* grid of a 2D map.

Amperometric operation was performed using platinum microdisk of 10 μm diameter. The electrochemical cell was completed with a Pt wire counter electrode, and an Ag/AgCl/KCl (sat.) as the reference electrode. All potential values reported in this work are referred to the Ag/AgCl/KCl (sat.) reference electrode. Amperometric measurements were performed using two SECM operation modes, namely the Surface Generation/Tip Collection (SG/TC) and the Redox Competition (RC) modes. In the SG-TC mode, electroactive species released from the surface are oxidized or reduced at the tip. The currents associated with these reactions can be correlated with the electrochemical activity of the sites on the surface [37]. In this work, the SG/TC mode was employed to study the evolution of hydrogen gas (H_2_) from a corroding surface when exposed to 5 mmol L^−1^ NaCl solution. H_2_ evolution from the surfaces of different metals has been investigated by SECM in the SG/TC mode which allows their localized corrosion processes to be studied [38,39,40,41]. These previous studies reported the selective detection of reacting species released from the corrosion reactions when the materials are left unpolarized in the environment. Good sensitivity and resolution towards hydrogen oxidation were achieved when the Pt probe was biased at 0.0 V, and therefore the SECM studies using SG/TC mode were employed with the Pt probe biased at this potential with Reaction (1) occurring at the tip:

H_2_ → 2H^+^ + 2e^−^ (1)

On the other hand, the redox competition mode was employed to monitor the molecular oxygen content in the solution through its electro-reduction at the tip in aerated electrolytes at neutral and alkaline pH. By choosing a tip potential in the range between −0.60 and −0.90 V the electrochemical responses associated with the reduction of oxygen can be investigated [42]. In this work, SECM operation in the redox competition mode was performed with the Pt probe biased at −0.70 V for Reaction (2),

O_2_ + 2H_2_O + 4e^−^ → 4OH^−^ (2)

Since this reaction can also occur at the investigated substrate in the event of corrosion through the local microcells developed on the surface of the material, competition for the oxygen dissolved in the solution will be established between the Pt microelectrode and the cathodic sites on the corroding surface [43]. Changes in the amount of current measured at the Pt microelectrode as result of such competition are the basis for this operation mode of SECM.

The tip-sample distance was set at 20 μm after recording *Z*-approach curves over the surrounding resin with the Pt polarized at −0.70 V to use the reduction of dissolved oxygen in the electrolyte as redox mediator under quasi-diffusion-controlled conditions (Reaction (2)). Thus, a variation in the profile of the limiting current was observed near the surface (insulating resin) while the Pt microelectrode monitored the electro-reduction of the dissolved oxygen, thus detecting the location of the surface. Subsequently, SECM scans were acquired in constant height by scanning the Pt tip in the *XY* plane parallel to the investigated surfaces.

Potentiometric operation was attained using antimony microelectrode tips [23]. They were prepared from micropipettes obtained by pulling borosilicate capillaries (outside Ø = 1.5 mm, wall thickness Ø = 0.225 mm, obtained from Hilgenberg GmbH, Malsfeld, Germany) using a model P-30 micropipette puller (Sutter Instrument, Novato, CA, USA). Antimony fibre (approximately 2.0 cm length and approximate 15–20 μm diameter) was introduced into the lumen of the micropipette with the tip reaching out about 20 mm. Liquid mercury metal and copper wire (12 cm length and 0.5 mm dia.) were used to provide electrical contact. Loctite^®^ adhesive was used to seal the two micropipette ends. More details and information on the fabrication of Sb microelectrodes are described elsewhere [23]. A voltage follower based on a 10^13^ Ω input impedance operational amplifier (TL071, Texas Instruments, Dallas, TX, USA) was introduced in the measuring circuit. Calibration of the antimony tip was performed using a series of buffer solutions covering the 3 ≤ pH ≤ 11 range. A good linear relationship between the potential and the solution pH was observed in this pH range, with a slope of −42 mV per pH unit. A sub-Nernstian response is obtained instead of −59.5 mV per pH unit, which is a common finding for pH-sensitive antimony microelectrodes based on polycrystalline Sb/Sb_2_O_3_ [21,23,26,28].

For the potentiometric measurements, the antimony microelectrode was used in combination with an Ag/AgCl/KCl (sat.) reference electrode, constituting the electrochemical cell. The probe was placed at a height of approximately 50 μm above the investigated surface, which was adjusted and monitored with the help of a video camera TV system.

## 3. Results and Discussion

### 3.1. SECM Characterization of a Model Mg-Al Galvanic Pair

The amperometric and potentiometric SECM probes were used to characterize a Mg-Al galvanic pair immersed in naturally aerated NaCl solution, using a combination of amperometric and potentiometric operation modes in SECM. Amperometric and potentiometric SECM operations were initially performed on a model Mg-Al galvanic pair system in order to check the efficiency of these probes in the characterization of a galvanic pair system similar to that occurring in the spontaneously corroding 2098-T351 aluminum alloy. Conventionally, galvanic corrosion occurs when two dissimilar metals are connected electrically at the rear of the mold containing the metal samples, while they are exposed to the test solution at the top side. By using a model system, the anodic and cathodic reactions are expected to be spatially separated over the two metal surfaces. That is, the electric coupling will originate preferential and accelerated dissolution of the less noble metal acting as the anode of the corrosion cell, while the cathodic reaction process will be associated with the more noble metal [21].

**Figure 1:**
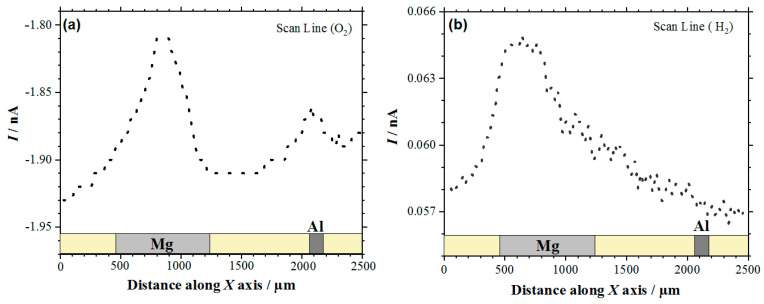
Line scans recorded by scanning electrochemical microscopy (SECM) with the Pt tip travelling above the model Mg-Al galvanic couple immersed in 5 mmol L^−1^ NaCl solution for approximately 1 h. Amperometric SECM mode: (**a**) Redox competition (RC) for the reduction of O_2_ (Pt tip biased at −0.70 V), and (**b**) Surface Generation/Tip Collection (SG/TC) for the oxidation of hydrogen gas evolving from the sample (Pt tip biased at 0.0 V). Tip-substrate distance: 20 μm; scan rate: 100 μm s^−1^.

The visualization of the Mg-Al galvanic couple was first attempted by recording line scans across the two metals with the Pt microelectrode, sequentially polarized at −0.70 and 0.0 V. In this way, the reduction of dissolved oxygen and the oxidation of hydrogen gas could be monitored with spatial resolution, thus allowing for the anodic and cathodic reactions occurring in the galvanic pair to be identified on these electrically-connected metals. Figure 1 shows typical line scans recorded after ca. 1 h immersion in the electrolyte. It must be noted that, for the sake of comparison, the line scan recorded with the tip biased at −0.70 V for the electro-reduction of molecular oxygen has been plotted with decreasing tip currents along the *Y* axis (cf. Figure 1a), whereas direct plotting has been chosen for the line scan depicted in Figure 1b. In this way, and for the two line scans, the location of the galvanic corrosion reactions can be detected as current peaks from the heterogeneously-distributed tip currents that are measured while the tip was travelling over the sample.

**Figure 2:**
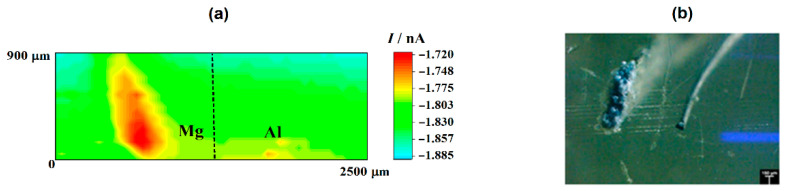
(**a**) SECM map obtained with the Pt tip travelling above the model Mg-Al galvanic couple immersed in 5 mmol L^−1^ NaCl solution for approximately 2 h. The image was obtained using the RC mode for the reduction of O_2_. Tip biased at −0.70 V; tip–substrate distance: 20 μm; scan rate: 75 μm s^−1^. (**b**) Optical micrograph of the model Mg-Al galvanic couple spontaneously corroding after 2 h immersion in 5 mmol L^−1^ NaCl solution.

In the case of the RC mode, oxygen depletion was observed above the two metals, although it occurred in a lesser extent on aluminum than on magnesium (cf. Figure 1a). Since the tip-substrate distance was maintained constant during the measurements, the near field effect on oxygen diffusion towards the tip that arises from the proximity of the sample was attained over the resin between the two metals and on the opposite ends of the line scan. Accordingly, stationary tip currents were expected to be observed, when the tip passed over the insulating resin. However, the occurrence of some small unavoidable tilt might be deduced by ideally taking a baseline for the currents measured when the tip was travelling over the resin areas, showing a smaller tip–substrate distance towards the right of the graph. The occurrence of measurable depletions in the molecular oxygen concentration in the electrolyte volume comprised between and the scanned surface directly correlates with the detection of cathodic sites in the corroding system as oxygen consumes the electrons released by the metal, thus leading to competition between the probe and the corroding surface for the reduction of this molecule. Although smaller reduction current values were measured at the tip when passing above the two metals, the current decrease was three to four times smaller above magnesium. This feature may seem quite surprising at first, since magnesium is not the more noble metal in the galvanic pair. Even further, the oxygen reduction reaction is known to not be the main cathodic process in this metal either [43]. Instead, hydrogen evolution is expected to dominate the cathodic process (Reaction (1)). Thus, this observation effectively supports that more extensive cathodic activity must be occurring in this metal. On the other hand, the different area ratio between the two metals in the model galvanic pair used in this work must be taken in account, which may justify that the aluminum area is not big enough to provide sufficient cathodic sites for the consumption of the electrons left by the magnesium ions as they dissolve in the electrolyte. In the event of high magnesium dissolution rates, the electrons would preferentially be consumed in locations close to the anodic sites on magnesium, therefore reducing the contribution of cathodic sites developing on aluminum. These observations are confirmed by the inspection of the 2D SECM map shown in Figure 2a. In this case, the complete exposed areas of the two metals are scanned by the Pt microelectrode tip after approximately 2 h immersion in the test electrolyte. Oxygen depletion occurred next to both metals, although it occurred to a greater extent over the surface of magnesium because the amount of oxygen available over the Al substrate was bigger. For the sake of easier identification, Figure 2b shows an optical micrograph of the exposed sample, facilitating distinguishing between the location and different area ratios of the two metals in the model galvanic couple.

The cathodic behavior of Mg, when galvanically coupled to more noble metals such as Cu and Al, was already observed by Snihirova et al. [44]. According to these authors, oxygen consumption was observed in the vicinity of an active Mg surface which was coupled to Cu and Al samples. Although higher oxygen consumption occurred mainly on the Cu surface—as it was expected—it was also observed on the Mg surface. The oxygen depletion over Mg was caused by its cathodic activity. The pH mapping and the reduction of dissolved oxygen clearly showed the occurrence of cathodic processes over the Mg substrate.

Next, the cathodic processes developed on the Mg surface also led to hydrogen gas (H_2_) evolution on this surface, as noted by the line scans in Figure 1b and the optical micrograph in Figure 2b. In the line scan depicted in Figure 1b it is possible to observe H_2_ evolution on the Mg surface by using the SECM in the SG/TC operation mode. Conversely, H_2_ evolution was not observed on the Al substrate. Therefore, in this study the Mg surface acts as the location of cathodic sites in the galvanic corrosion process. Finally, by comparing the shapes of the current peaks shapes in the two line scans of Figure 1 that are found above the magnesium sample, it seems that the oxygen reduction reaction occurs over a smaller region than the evolution of hydrogen over this metal. This observation will be further discussed below.

After the amperometric SECM measurements were performed on the Mg-Al galvanic pair, potentiometric SECM operation was performed using a Sb/Sb_2_O_3_ microelectrode tip. As seen previously, metal dissolution occurs at the anodic sites, whereas oxygen consumption and hydrogen evolution occurs at the cathodic sites. As indicated by Reaction (2), localized formation of hydroxyl anions at the cathodic surface had to occur around the cathodic sites due to the oxygen reduction reaction, thus shifting the local pH in the alkaline direction. Furthermore, hydrogen evolution from water electrolysis at the cathodic sites on magnesium produce hydroxyl anions too:

2H_2_O + 2e^−^ → H_2_ + 2OH^−^ (3)

**Figure 3:**
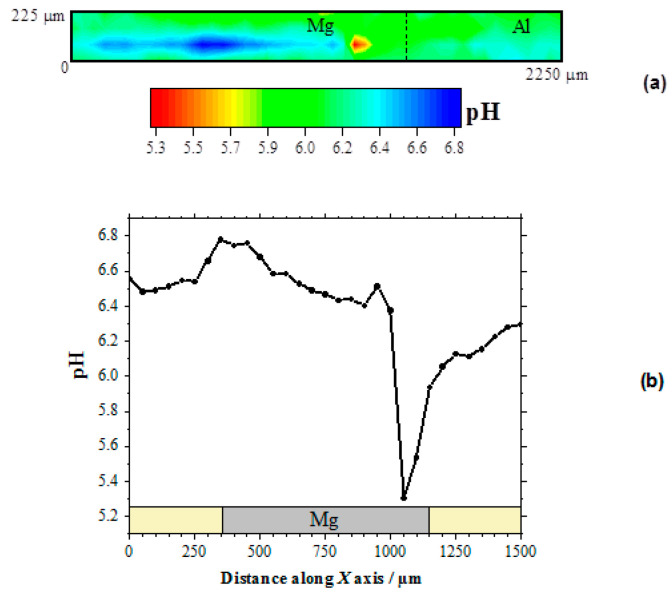
pH distribution above the model Mg-Al galvanic couple immersed in 5 mmol L^−1^ NaCl solution for approximately 2 h. The SECM data were obtained using an Sb/Sb_2_O_3_ microelectrode tip in potentiometric operation. (**a**) 2D map covering the model Mg-Al galvanic system; (**b**) line scan recorded over the horizontal symmetry axis of the Mg sample. Tip–substrate distance: 50 μm; scan rate: 80 μm s^−1^.

Therefore, the local pH changes produced in the electrolyte near the corroding galvanic Mg-Al couple will provide information regarding the distribution of the corroding microcells developed on the system. Figure 3a shows the pH distribution 2D-image obtained above the galvanic pair. In this pH map, the blue scale color indicates the areas with higher pH values than in the bulk of the electrolyte. In the 2D-map, this region is observed in the proximity of the Mg surface, and only a small and weak alkalinization might be observed in the proximity of the aluminum wire. On the other hand, the red scale color represents the area with lower pH, which was also observed on the Mg substrate though at a separate location. No evidence of electrolyte acidification occurred in the proximity of aluminum, thus evidencing that the anodic processes only occurred on the magnesium surface. Thus, both local alkalinization and local acidification were observed over the surface of Mg, indicating that highly localized metal dissolution occurred on a portion of the exposed metal surface, whereas the remaining metal was electrochemically active for sustaining the cathodic processes that consumed the electrons released by the dissolving metal ions. The proximity and greater surface area available on the magnesium ribbon compared to the aluminum wire in our particular model Mg-Al galvanic couple, led to the preferential localization of the cathodic activity on the magnesium ribbon. The complete sequence of pH changes occurring over the magnesium strip due to galvanic coupling to aluminum is better resolved by extracting a line scan along the horizontal axis of the metal, as shown in Figure 3b.

The occurrence of both cathodic and anodic sites on the magnesium ribbon was thus observed. Indeed, locations with pH values both higher and lower than those in the bulk electrolyte are observable in this line scan. As expected, alkalinization occurred around the cathodic sites, whereas local acidification is correlated with the development of anodic sites on the surface of the magnesium sample. In fact, magnesium dissolution occurs in competition with metal hydrolysis, as described by Reaction (4), thus contributing to local acidification at the anodic site:

Mg^2+^_(aq)_ + H_2_O → Mg(OH)^+^ + H^+^ (4)

Additional hydrolysis of Mg(OH)^+^ to Mg(OH)_2_ could occur, leading to the precipitation of corrosion products near the anodic sites, although it is quite unlikely due to the high acidification produced around a propagating pit. Instead, further acidification can result from the formation of hydroxyl-chloride complexes, a process typically accompanying hydrolysis in chloride-containing corrosion media, effectively shifting the hydrolysis reaction (Reaction 4) further to the right. Thus, H^+^ ions present on the Mg substrate cause a local reduction in the pH, and local acidification is the result of the hydrolysis reactions of the magnesium ion. The pH changes related to both the cathodic process and the hydrolysis by dissolved metals in corrosion systems are described in the literature [29,30].

Further inspection of the line scan in Figure 3b evidences the much more localized character of the region presenting acidification than that with the higher pH values. In addition, the pH gradient is higher for the anodic site as well. This is an indication that the anodic reaction occurred in a more localized manner, leading to the formation of corrosion pits on the corroding surface, whereas the cathodic reaction was more distributed over the remaining metal surface without effectively producing changes in the surface topography at the cathodic sites. The sharpness of the corroding pit that is observed as the tip approached from the left could be expected to apply at the opposite side as well. Yet the shape of the pH valley appears to be less sharp at the right side, but it should be realized that the observed broadening resulted from convective effects produced by the tip as it moved towards the right side and it could have carried some acid electrolyte with it. Next, the slight alkalinization observed around 950 μm on the *X* axis, i.e., near the left edge of the growing pit, must arise from the reduction of water in a cathodic site, according to Reaction (3). On the other hand, the lowest pH value recorded in this line scan, namely 5.3, is consistent with the maximum acidification expected for the hydrolysis of magnesium in a confined volume such as the conditions occurring within a propagating pit [19]. It must be noted, that pH values more acidic by 1–2 units would be expected in the case of the hydrolysis of aluminum ions [19], thus effectively confirming that the anodic sites occur solely on the magnesium sample for this model Mg-Al galvanic couple. Furthermore, the SECM results described in this section indicate that the cathodic process occurs preferentially on the surface of magnesium, leading to the evolution of hydrogen gas and the reduction of dissolved oxygen, although the latter scarcely occurs in proximity of the anodic sites, and it is observed to occur at further distances on magnesium as well as on the aluminum wire.

### 3.2. SECM Characterization of the 2098-T351 Al-Alloy

After the localized distribution of anodic and cathodic sites on a model Mg-Al galvanic couple was resolved in situ by SECM using a combination of amperometric and potentiometric probes, these probes were employed to characterize the electrochemical behavior of 2098-T351Al-alloy immersed in the same test solution. No polarization was applied to the metallic substrate, therefore any electrochemical reactivity would arise from the spontaneous development of corrosion processes on the surface of the alloy due to chemical heterogeneities in the system.

**Figure 4:**
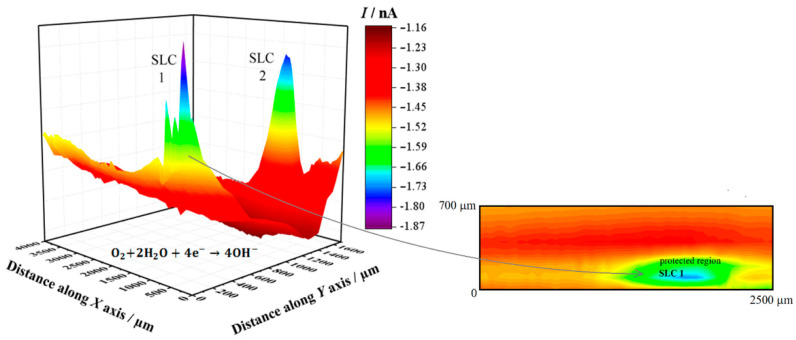
SECM map obtained with the Pt tip travelling above 2098-T351 immersed in 5 mmol L^−1^ NaCl solution for approximately 3 h. The image was obtained using the RC mode for the reduction of O_2_ (Pt tip biased at −0.70 V). The 2D map at the right side shows a magnification of the region around the feature labelled SLC 1 in the 3D map. Tip–substrate distance: 20 μm; scan rate: 100 μm s^−1^.

Figure 4 shows the SECM map recorded after approximately 3 h exposure of the alloy to 5 mmol L^−1^ NaCl solution. The image was obtained using the RC mode for the reduction of O_2_ (Pt tip biased at −0.70 V). In this way, it was possible to observe the electrochemical activity associated with the electro-reduction of oxygen recorded on the corroding alloy. Here, the upper and lower color scales are an indication of different tip current intensities for the electro-reduction of oxygen. The scale range corresponding to the upper scale color (i.e., red) represents the areas on the surface that originated from the greater oxygen consumption in the adjacent electrolyte volume, effectively indicating regions with depletion of oxygen in the adjacent electrolyte. The 2098-T351 surface shows regions (sites) with higher oxygen reduction currents (low oxygen depletion) in comparison with the remaining surface. In this SECM mode, a redox-competition process between the corroding surface and the tip occurs for the electro-reduction of oxygen in solution [42,45]. That is, oxygen can be consumed both at the tip and the surface, resulting in competition for this species. Therefore, if regions with lower reduction currents are associated with higher oxygen depletion, the decrease in the reduction current at the tip thus corresponds to oxygen depletion at the probe. This behavior was associated with the development of a cathodic region in Section 3.1. A heterogeneous distribution of oxygen reduction currents was monitored at the tip during the SECM measurement shown in Figure 4, indicating variations in oxygen availability over the alloy surface. The reduction current increased locally when the tip passed over the SLC sites, indicating a greater availability of oxygen dissolved in the electrolyte at those locations. Additionally, oxygen consumption mainly occurred over the rest of the surface. Consumption of oxygen in the proximity of the SLC sites was also observed, nevertheless, this was significantly smaller than that observed on the remaining surface at which SLC sites were not developed.

**Figure 5:**
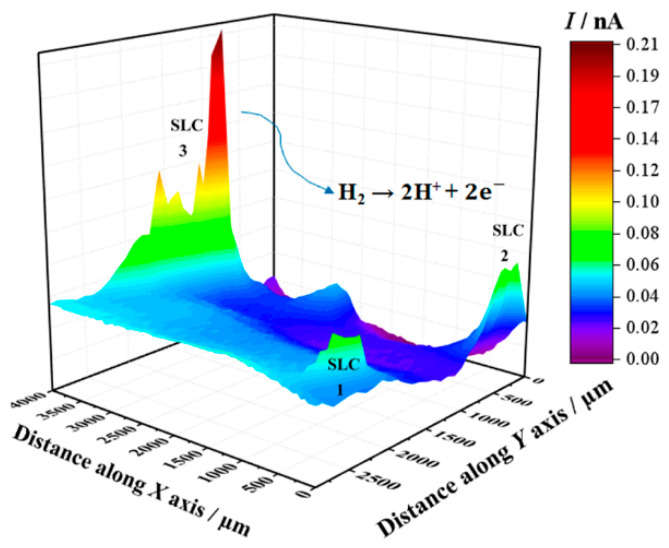
SECM map obtained with the Pt tip travelling above 2098-T351 immersed in 5 mmol L^−1^ NaCl solution for approximately 3 h. The image was obtained using the SG/TC mode for the oxidation of hydrogen gas evolving from the alloy (Pt tip biased at 0.0 V). Tip-substrate distance: 20 μm; scan rate: 100 μm s^−1^.

Next, SECM maps of the 2098-T351 surface were recorded to monitor the electrochemical response associated with H_2_ evolution on the spontaneously corroding alloy using the SG/TC mode. In this way, the evolution of molecular hydrogen (H_2_) was effectively sensed at the tip through an oxidation reaction as described above (according to Reaction (1)). In the SECM map shown in Figure 5, the upper and lower color scales correspond to different tip current intensities due to the oxidation of hydrogen gas generated at the corroding 2098-T351 surface. The upper scale colors (red and yellow/green) indicate the areas with high H_2_ evolution rate, whereas the lower scale colors represent those surface regions without H_2_ evolution. It is interesting to note, that the cathodic reaction related to hydrogen evolution occurs solely at very localized sites on the surface, thus related to the second phases present on the surface. That is, although the aluminum-rich metal matrix can serve as the site for the cathodic reactions to occur on the surface, this substrate is unable to support the hydrogen evolution reaction on its remaining surface, where SLC sites were not developed.

**Figure 6:**
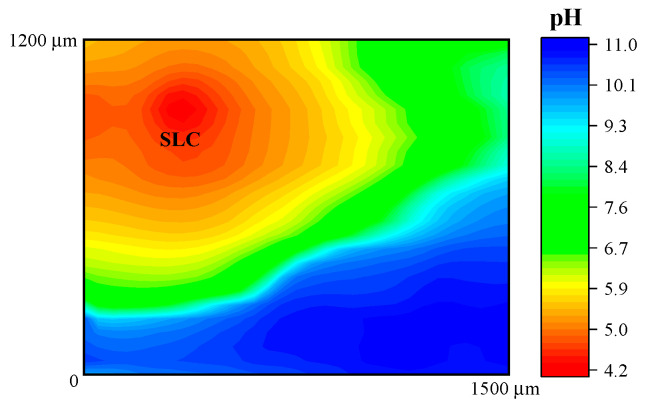
pH distribution above 2098-T351 immersed in 5 mmol L^−1^ NaCl solution for approximately 3 h. The image was obtained using an Sb/Sb_2_O_3_ microelectrode for potentiometric SECM operation. Tip–substrate distance: 50 μm; scan rate: 80 μm s^−1^.

In addition, Figure 6 shows the response obtained when the potentiometric probe was used to characterize the 2098-T351 Al-alloy. The sample was immersed in NaCl solution using the Sb/Sb_2_O_3_ microelectrode in order to observe the pH distribution over the corroding alloy. As described above, the cathodic reaction occurring in naturally-aerated solutions is the reduction of dissolved oxygen in the electrolyte according to Reaction (2), which leads to local alkalinization of the solution at the cathodic sites. Thus, in Figure 6, the red scale color represents the areas with lower pH that is associated with the SLC sites, whereas the blue scale color indicates the areas with higher pH (no SLC sites developed) on the 2098-T351 surface. Thus, local acidification above the SLC regions and alkalinization above remaining corroding alloy were observed.

**Figure 7:**
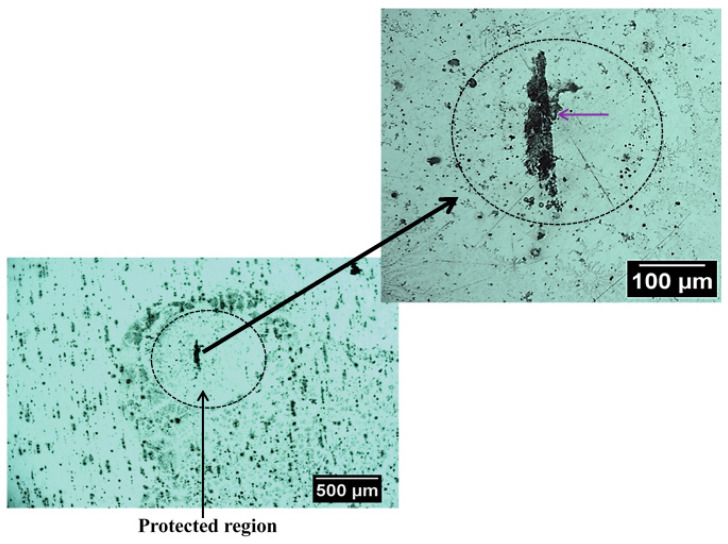
Optical micrographs showing a region with severe localized corrosion (SLC) that was developed on the 2098-T351 surface after immersion in 5 mmol L^−1^ NaCl solution for approximately 3 h.

Figure 7 shows an optical image of the surface of this alloy that exhibit SLC site after immersion in NaCl solution. It is possible to observe localized corrosion sites over the surface which are usually associated with micrometric constituent particles, also called trenching, and severe localized corrosion (SLC) sites [46,47]. As mentioned earlier, the Al-Cu-Li alloys are susceptible to localized corrosion processes, which are associated with the presence of hexagonal T1 phase [10,11,12]. The T1 phase has been reported to be the phase responsible for the development of SLC in Al-Cu-Li alloys [48,49,50,51,52]. Since the 2098-T351 Al-Cu-Li alloy contains T1 phase, regions with higher densities of these phases should be more susceptible to SLC [7,8]. Therefore, two distinct corrosion mechanisms can be observed on the 2098-T351 Al-alloy because the SLC sites present deeper attack compared to the trenching sites related to micrometric particles, where the attack is more superficial [2,53]. Accumulation of corrosion products on the surface of the alloy should occur gradually in regions near the SLC sites and more intensely in those regions outside the SLC sites associated with the micrometer particles, since the attack in these regions (outside the SLC sites) occurs due to local alkalinization related to the cathodic nature of the micrometric particles.

The SLC has been associated with large and deeper corroded areas that develop in Al-Cu-Li alloys, which also present a cathodically protected region, development of a surrounding corrosion product ring, and evolution of hydrogen bubbles generated inside the SLC site [54,55]. The main reactions involving the formation process of SLC are given by:

Al → Al^3+^ + 3e^−^ (5)

Al^3+^ + 3H_2_O → Al (OH)_3_ + 3H^+^ (6)

2H^+^ + 2e^−^ → H_2_↑ (7)

During the corrosion process, the oxidation of Al to Al^3+^ occurs in the anodic regions (Reaction (5)), and the hydrolysis by the dissolved metal ions releases hydrogen ions (Reaction (6)), that results in a decrease in pH inside the site, causing the local acidification in the close proximity of the SLC region. Concomitantly, this process favors the evolution of hydrogen gas on the surface (Reaction (7)). Thus, when the amperometric tip (polarized to 0.0 V) passes over the SLC site, it promotes the oxidation of the generated H_2_ gas at the cathodic regions close to the SLC, as given by Reaction (2). Therefore, since hydrogen evolution is a primary feature of the anodic SLC site, the SECM map given in Figure 5 shows the activity of the SLC sites. For the aluminum based alloys, hydrogen evolution is associated with the anodic region due to an autocatalytic reaction inside the pits that favors the production of H^+^ and its reduction in the cathodically protected area just outside the pit. Furthermore, the formation of a cathodically protected area, due to electron migration in the vicinities of a SLC site (cf. Figure 7), thus favors the reduction of O_2_ according to Reaction (2). As a consequence, the production of OH^−^ outside the pit, in addition to the release of Al^+3^ ions undergoing dissolution, there is an occasion for the precipitation of the corrosion product Al(OH)_3_ around the SLC region (according to Reaction (6)). This explains the ring-like protected region observed in Figure 7.

In this context, is important to note that, although the reduction of H^+^ to produce H_2_ happens inside the SLC site, H^+^ ions are also reduced around the SLC site, thus leading to the migration of hydrogen ions from the interior of the SLC site to the SLC mouth under an electrostatic potential difference [45]. The development of a boundary region between the lower pH region at the SLC sites and the higher pH region surrounding these sites was imaged in the potentiometric SECM map given in Figure 6.

Despite the possibility of oxygen reduction just outside the SLC region, the SECM image showed in Figure 4 indicates that oxygen consumption was predominant in the region on the surface away from the SLC region. This is explained by the higher amount of micrometric particles, predominantly composed of Al, Cu and Fe [2,3]. These micrometric particles are cathodic in regards to the Al matrix and, therefore, the reduction reaction that consumes oxygen must occur over these particles [53,56]. Due to the higher anodic currents associated with the SLC, higher reduction currents are observed outside the SLC. Micrometric particles were also observed close to the SLC sites, however, the precipitation of corrosion products, as a consequence of local alkalinization, is more extended over the micrometric particles outside the SLC region (see Figure 7). Therefore, the consumption of most electrons released by Al dissolution occurs over the micrometric particles outside the SLC site in the oxygen reduction reaction. This result is in agreement with previously published works [57,58].

In summary, on the basis of the results obtained in this work, it was possible to characterize the electrochemical behavior of the 2098-T351 Al-Cu-Li alloy. According to SECM characterizations, the main anodic sites were associated with the SLC regions. Anodic dissolution of the Al and hydrogen evolution was observed at these sites. On the other hand, the main cathodic reaction was identified to be the reduction of dissolved oxygen, which spreads over the micrometric particles in the remaining surface.

## 4. Conclusions

In this work, the local distribution of anodic and cathodic sites in the corroding surface of the 2098-T351 Al-Cu-Li alloy was performed using an adequate combination of operation modes in the scanning electrochemical microscope (SECM). Pt and Sb/Sb_2_O_3_ microelectrodes were employed as probes for amperometric and potentiometric operations, respectively.

Local distributions in electrochemical activity and pH were associated with the development of localized anodes and cathodes on the surface of the alloy were imaged in situ using these SECM probes.

In an initial study performed on a model Mg-Al galvanic pair, it was possible to test the response of these probes to characterize a galvanic pair condition. Local alkalinization and higher oxygen consumption related to cathodic activity, were detected above the surface of magnesium and anodic activity leading to local acidification occurred at the aluminum substrate.

The probes were subsequently employed to characterize the 2098-T351 Al-alloy. Changes in the concentration of oxygen dissolved in the electrolytic phase, H_2_ evolution and local pH were imaged with high resolution, and they were satisfactorily related to the development of cathodic and anodic regions on the alloy surface. The sites of severe localized corrosion (SLC) on the 2098-T351 surface were the most anodic regions compared to remaining surface. Higher hydrogen evolution, lower oxygen consumption and higher acidity were observed on the SLC sites.

It was demonstrated that amperometric and potentiometric probes are useful for the furtherance of the understanding of the localized corrosion and electrochemical activities of Al-Cu-Li alloy.

## Data Availability

The data presented in this study are available in the current work.
